# Autophagy Meets Aging: An Overview

**DOI:** 10.3390/cells12030489

**Published:** 2023-02-02

**Authors:** Anna Picca, Emanuele Marzetti, Christiaan Leeuwenburgh

**Affiliations:** 1 Department of Medicine and Surgery, LUM University, 70100 Casamassima, Italy; 2 Fondazione Policlinico Universitario “A. Gemelli” IRCCS, 00168 Rome, Italy; 3 Department of Geriatrics and Orthopedics, Università Cattolica del Sacro Cuore, 00168 Roma, Italy; 4 Department of Physiology and Aging, University of Florida, Gainesville, FL 32610, USA

Aging is characterized by biological disarrangements that increase vulnerability to stressors, the development of chronic diseases (e.g., diabetes, cardiovascular disease, cancer, neurodegeneration), and functional decline. Alterations in autophagy and other cellular quality control systems have recently been included among the processes underlying aging and associated disease conditions [1]. Compromised autophagy fails at recycling obsolete cell components and contributes to the accumulation of intracellular protein aggregates, damaged mitochondria, and lipofuscin deposits. Organelle-specific autophagy pathways (including mitophagy, which selectively targets mitochondria) have also been identified [2,3]. Intracellular components may hold pro-inflammatory properties; therefore, a coordinated activity of recycling machineries is especially relevant during aging to limit the bulk release of damage-associated molecules and halt inflamm-aging [3,4]. If the molecular determinants of these changes were unveiled, innovative anti-aging remedies and personalized interventions targeting cellular quality control could be developed to extend health- and lifespan.

In the Special Issue, titled “Autophagy meets aging II”, we collected contributions investigating changes in autophagy and other cellular quality control processes in the context of aging and associated conditions. This Special Issue is a second edition of the editorial project “Autophagy meets aging” that gathered contributions from different, yet complementary, points of view by clinicians and basic researchers working in the field of biogerontology in humans and pre-clinical models [5,6,7,8,9,10,11,12].

Altered autophagy and mitochondrial dysfunction have been reported to arise following ischemia/reperfusion (I/R) in liver, leading to reduced hepatocyte survival [6]. This was partly ascribed to the dysregulated activity of the endogenous calpain inhibitor calpastatin (CAST) that is activated by I/R and triggers hepatocyte death [6]. The analysis of liver biopsies from aged mice and humans clarified that the reduced tolerability of aged liver to I/R was associated with the diminished intrinsic half-life of CAST after I/R. Therefore, CAST depletion has been proposed as a contributor to age-related liver injury following I/R [6].

Sarcopenia, the decline in muscle mass and strength that occurs with age, has also been associated with altered autophagy [10,13]. Defective mitochondrial tagging and removal has been implicated in the set of events leading to muscle atrophy and physical function decline because of inefficient energy generation and the increased production of reactive oxygen species. The clearance of mitophagy-engulfed cargoes has been indicated as a pathway that may be exploited to develop therapeutics against sarcopenia [10]. The relationship between mitophagy markers, measures of physical performance, and tissue composition of the lower extremity was investigated in physically inactive older adults [14]. Smaller muscle volume and lower tissue composition indices were found in older adults compared with younger participants, suggesting that altered mitophagy may be associated with the deterioration of lower extremity tissue composition and muscle dysfunction [14]. Mitochondrial alterations have also been linked with perturbations in iron homeostasis in the muscles of older people with low physical performance [8]. The analysis of protein levels of iron transporters (i.e., mitoferrin and frataxin), mitochondrial DNA (mtDNA) deletion, and autophagy/mitophagy markers in muscle samples from physically inactive older adults unveiled perturbations in cellular and mitochondrial iron homeostasis [8]. Taken as a whole, these findings support the hypothesis that age-related derangements in mitochondrial quality control processes may contribute to mtDNA instability [8]. 

The accrual of neurotoxic misfolded proteins in motor neurons due to inefficient autophagy clearance has been recognized as a pathological feature of amyotrophic lateral sclerosis (ALS) and a trigger of neurodegeneration. The upregulation of autophagy via pharmacological agents has been shown to promote the clearance of protein aggregates and alleviate disease phenotypes in vitro and in vivo [12]. However, further study is needed to test the therapeutic strategies targeting autophagy in ALS [12].

Altered autophagy and lipophagy has been described in the fibroblasts of patients carrying the p.K126R RAB7 mutation responsible for the Charcot–Marie–Tooth type 2B disease, a dominant axonal peripheral neuropathy [5]. Patients with K126R mutations showed a concomitant increase in lysosomal activity and autophagy that was associated with a motor phenotype [5]. Conversely, an age-associated increase in the protein expression of the autophagy inhibitor Rubicon was observed in postmortem brains and human-induced pluripotent stem cells of people with Alzheimer’s disease (AD), as well as in transgenic mice and neuroblastoma cells [15]. Of note, the Rubicon protein was found to be localized at the level of neurons and implicated in neurodegeneration via the accumulation of toxic protein aggregates [15]. Transgenic AD mice lacking the protein Rubicon showed accrual of the amyloid β protein within the hippocampus and a lower expression of the autophagy receptor p62 [15]. Accordingly, neuroblastoma cells expressing the amyloid precursor protein (APP) showed higher APP/amyloid β secretion in the absence of Rubicon, which was not observed in cells lacking autophagy-related protein 5 or Ras-related protein 27, a regulator of exosome secretion [15]. These findings suggest a role for Rubicon in the amyloid pathway and a possible new therapeutic target for AD and other neurodegenerative disorders [15].

A decline in neuronal autophagy has also been implicated in determining lifespan and healthspan [11]. Premature cell death has been reported in the ascomycete Podospora anserina, depleted of PaATPE, an assembly factor, of mitochondrial F1Fo-ATP-synthase [7]. PaATPE is pivotal for determining the shape and maintenance of mitochondrial ultrastructure, which impacts the organelle function, oxidant generation, and programmed cell death [7]. The deletion of PaAtpe and PaAtg1 genes, encoding key components of the autophagy machinery, led to reduced mitophagy and lifespan restoration in Podospora anserina [7]. These data suggest an altered F1Fo-ATP-synthase dimerization is involved in autophagy-induced cell death.

Cellular resilience has been shown to be achieved via the cyclin-dependent kinase inhibitor p27Kip1 that promotes autophagy and inhibits apoptosis. The functions of p27Kip1 vary according to its localization. While the cytosolic isoform of p27Kip1 promotes cellular resilience, nuclear p27Kip1 can inhibit cell cycle progression and trigger apoptosis and/or cellular senescence [9]. The differential localization of p27Kip1 is regulated by kinase-dependent phosphorylation, including protein kinase B (AKT) and 5′ AMP-activated protein kinase (AMPK). The nuclear relocation of p27Kip1 is enhanced during aging [9].

The follicle-stimulating hormone (FSH), a well-known pro-surviving follicular growth factor, has been shown to alleviate the effects of aging on granulosa cells cultured in d-galactose-enriched media [16]. This phenomenon seems to occur via the activation of mitophagy and AMPK signaling. Enhanced glycophagy was found in d-gal senescent cells, an effect that was reinforced by FSH treatment and further promoted via the activation of phosphoinositide 3-kinases (PI3K)/AKT [16]. Notably, PI3K and AKT inhibitors (LY294002 and GSK690693) alleviated the effects of FSH on glycophagy. The simultaneous blockade of AMPK and PI3K/AKT pathways blunted the positive effects of FSH on energy modulation in ovarian cell senescence, suggesting that glycophagy and mitophagy via the PI3K/AKT and AMPK may be targeted for alleviating ovarian aging [16]. Finally, findings by Harhouri et al. [17] indicate that the promotion of autophagy via proteasome inhibitors might serve as a strategy for the management of progeroid syndromes.
